# Relationship between Dermatology Life Quality Index Scores and EQ-5D-5L Utility Values in Adults with Atopic Dermatitis: Development of a Swedish Mapping Model

**DOI:** 10.2340/actadv.v106.adv-2025-0277

**Published:** 2026-06-02

**Authors:** Alexandra Metsini, Linda Ryen, Scott Montgomery, Anu Molarius, Åke Svensson, Laura Kobyletzki

**Affiliations:** 1 School of Medical Sciences, Faculty of Medicine and Health, Örebro University, Örebro, Sweden; 2 Department of Healthcare, Knowledge Support Unit, Region Värmland, Karlstad, Sweden; 3 University Healthcare Research Center, Faculty of Medicine and Health, Örebro University, Örebro, Sweden; 4 Clinical Epidemiology and Biostatistics, School of Medical Sciences, Faculty of Medicine and Health, Örebro University, Örebro, Sweden; 5 Clinical Epidemiology Division, Department of Medicine, Solna, Karolinska Institute, Stockholm, Sweden; 6 Department of Epidemiology and Public Health, University College London, London, United Kingdom; 7 Centre for Clinical Research, Region Värmland, Karlstad, Sweden; 8 Department of Public Health Sciences, Karlstad University, Karlstad, Sweden; 9 Department of Dermatology and Venereology, Skåne University Hospital, Lund University, Malmö, Sweden; 10 Department of Occupational and Environmental Dermatology, Skåne University Hospital, Lund University, Malmö, Sweden

**Keywords:** atopic dermatitis, health-related quality of life, DLQI, EQ-5D-5L, mapping, patient-reported outcomes

## Abstract

Atopic dermatitis is associated with impaired health-related quality of life (HRQoL), especially in moderate-to-severe disease. The Dermatology Life Quality Index (DLQI) is the most widely used dermatology-specific measure, whereas the EuroQol Five-Dimension, Five-Level questionnaire (EQ-5D-5L), a generic preference-based instrument, assesses health status across diseases. This study examined the relationship between DLQI and EQ-5D-5L in Swedish adults with atopic dermatitis and developed a mapping model to estimate EQ-5D-5L utilities from DLQI scores. In this cross-sectional study, 112 Swedish adults with self-reported physician-diagnosed atopic dermatitis, recruited through the Swedish Asthma and Allergy Patient Association, completed both questionnaires. DLQI scores correlated strongly and negatively with EQ-5D-5L index values (*r*=−0.64, *p*<0.001). Each 5-point increase in DLQI corresponded on average to a 0.07 decrease in EQ-5D-5L utility. A fractional logit regression model including covariates age, sex and disease severity, showed good calibration and explained 27% of the variance in EQ-5D-5L values. These findings demonstrate that DLQI scores meaningfully reflect generic HRQoL among adults with atopic dermatitis and can be used to estimate EQ-5D-5L utilities when preference-based data are unavailable. The results enhance understanding of how DLQI relates to overall health status and support the application of DLQI-derived utilities in cost-effectiveness analyses and health technology assessments.

**SIGNIFICANCE**

This study quantifies how disease-specific and generic quality-of-life measures relate in adults with atopic dermatitis. Dermatology Life Quality Index (DLQI) and EuroQol Five-Dimension, Five-Level questionnaire (EQ-5D-5L) scores are strongly correlated, with poorer dermatology-specific quality of life corresponding to markedly lower general health status. The mapping model developed provides a basis for estimating EQ-5D-5L utilities from DLQI data, supporting future health economic evaluations. These findings also help clarify how DLQI outcomes relate to overall health status and highlight the significant impact of atopic dermatitis on daily life.

Atopic dermatitis (AD) is a chronic, relapsing inflammatory skin disease that typically begins in childhood and may persist or recur in adulthood, affecting around 10% of adults in developed countries (1, 2). It is characterized by intense pruritus, visible eczema and frequent sleep disturbance. AD is associated with impaired health-related quality of life (HRQoL) across physical, psychological and social domains, especially in moderate-to-severe cases (3, 4). It has also been recognized as the leading cause of global disability among dermatological diseases (5).

The burden of AD is commonly assessed using patient-reported outcome measures. The Dermatology Life Quality Index (DLQI) is the most widely used dermatology-specific instrument and provides a simple but robust evaluation of how skin disease affects daily life (6–8). In contrast, generic preference-based measures such as the EuroQol Five-Dimension, Five-Level questionnaire (EQ-5D-5L) (9) assess overall health status and are used for economic evaluations.

Because the DLQI is widely used in dermatology trials and registries, it is often the only HRQoL measure available. Mapping between DLQI and EQ-5D-5L allows estimation of preference-based utilities from dermatology-specific data, enabling cost-effectiveness analyses, health technology assessments and cross-disease comparisons.

Previous studies have demonstrated the feasibility of mapping dermatology-specific measures to generic preference-based utilities, confirming that DLQI can approximate health utilities. Ali et al. (10) analysed data from over 3,500 patients with various skin diseases across Europe, including eczema, and showed that DLQI could reliably predict EQ-5D-3L utilities with small mean errors (10). Vilsbøll et al. (11) focused specifically on AD, evaluating several models in more than 1,200 adult patients from Europe and the USA. Their best-performing regression mixture model, which included DLQI together with age and sex, accurately estimated EQ-5D-5L utilities (11).

Given potential cross-country differences in health perceptions, value sets and HRQoL norms, disease and country-specific mapping models are needed to ensure valid local interpretation and use in cost-effectiveness studies. As previous studies (10, 11) relied primarily on international samples with limited representation from Swedish patients, the aim of the present study was to examine the relationship between disease-specific quality of life, as measured by the DLQI, and general health status, assessed with the EQ-5D-5L, in Swedish adults with AD. In addition, the study sought to develop an exploratory mapping model to estimate EQ-5D-5L utility values from DLQI scores, thereby supporting both clinical interpretation and health economic evaluation of AD in Sweden.

## MATERIALS AND METHODS

### Study design and participants

This cross-sectional study was conducted in 2024 among adult members (aged ≥18 years) of the Swedish Asthma and Allergy Patient Association who reported physician-diagnosed AD. Participants were recruited through the Patient Association’s membership database and completed an online questionnaire including both the DLQI and the EQ-5D-5L. A total of 112 participants who completed both measures were included in this analysis. Further details on recruitment and survey procedures are described elsewhere (12), and additional methodological information is provided in the Appendix S1.

Ethical approval was obtained from the Swedish Ethics Review Authority (Dnr 2019–03720; amendment 2024-08-11). Written informed consent was obtained from all participants. The study was conducted in accordance with the Declaration of Helsinki (13). Data were pseudo-anonymized and handled in accordance with applicable Swedish data protection legislation and Good Epidemiological Practice.

### Measures

The DLQI is a 10-item dermatology-specific questionnaire assessing the impact of skin disease on daily life during the previous week (score range: 0–30, with higher scores indicating greater impairment) (6–8).

The EQ-5D-5L measures HRQoL across 5 dimensions (mobility, self-care, usual activities, pain/discomfort and anxiety/depression), each with 5 severity levels (9). Utility index values were derived using the Swedish EQ-5D-5L value set (14), which ranges from −0.314 (worse than death) to 1.0 (full health). Both instruments were completed concurrently.

In addition, participants reported demographic characteristics, itch and pain intensity, self-rated disease severity and allergic comorbidities, which are summarized in Table I. Self-rated disease severity was assessed using the Patient Global Assessment (PGA), a patient-reported 5-point response scale of overall AD severity, ranging from clear to severe, with demonstrated validity in adults with atopic dermatitis (15).

**Table I.** Descriptive statistics of the final study sample EQ-5D-5L health index score (−0.314–1)

Characteristics	Estimate
Respondents (*n*)	112
Age (mean±SD)	45.36±14.53
Median (IQR)	46.00 (23.00)
Women (proportion %)	87.50%
Disease severity *n* (%)	
Severe	10 (8.93)
Moderate	24 (21.43)
Mild	38 (33.93)
Clear/almost clear	40 (35.71)
Comorbid diseases *n* (%)	
Asthma	82 (54)
Allergic conditions	
Allergic rhinitis	72 (47)
Food allergies	98 (64)
Other allergies	106 (70)
Nonatopic conditions	
Depression	21 (14)
Anxiety	35 (23)
Eating disorders	5 (3)
ADHD	13 (9)
Other diseases	31 (20)
Itch intensity (0–10) (mean±SD)	4.98±2.55
Pain intensity (0–10) (mean±SD)	3.27±2.60
EQ VAS score (0–100) (mean±SD)	64.18±20.57
EQ-5D-5L health index score (−0.314–1), mean±SD	0.86±0.18
Median (IQR)	0.93 (0.13)
Min, max	0.07, 1
Kurtosis	9.60
Skewness	−2.50
DLQI (0–30), mean±SD	10.23±6.26
Median (IQR)	9 (9.00)
Min, max	1, 27
Kurtosis	2.91
Skewness	0.81

ADHD:attention-deficit/hyperactivity disorder; DLQI:Dermatology Life Quality Index; EQ-5D-5L:EuroQol Five Dimensions Five Levels; EQ-VAS:EuroQol Visual Analogue Scale; IQR:interquartile range; SD:standard deviation.

Further evidence supporting the content and construct validity of self-reported disease severity has also been reported (16). Responses were grouped into 4 categories for analysis: clear/almost clear, mild, moderate and severe. Additional details are provided in Appendix S1 and in a previous report (12).

### Mapping approach and statistical analyses

The mapping analysis was conducted in accordance with best-practice recommendations from the Mapping onto Preference-based Measures (MAPS) reporting standards (17), as well as guidance from the National Institute for Health and Care Excellence (NICE) Decision Support Unit (18) and the International Society for Pharmacoeconomics and Outcomes Research (ISPOR) (19). Analyses were conducted on complete cases with no missing data for either measure. Conceptual overlap between DLQI and EQ-5D-5L was assessed using Spearman correlation coefficients between total scores and between individual items and dimensions.

### Model selection

Candidate models were chosen based on their suitability for bounded utility data and consistency with mapping literature. Because EQ-5D-5L utilities fall within a fixed range, the modelling approach should account for their restricted distribution. Four approaches were evaluated.

- Ordinary Least Squares (OLS): a standard linear regression approach (20), commonly used as a baseline in mapping studies (10, 11);
- Tobit regression: accommodating potential censoring at the upper or lower bounds (21);
- Fractional logit generalized linear model (GLM-fl): appropriate for values within [0,1], addressing ceiling effects (22–24);
- One-inflated beta regression (OIBR): explicitly modelling the mass at full health (25, 26).

Given the modest sample size, more complex mixture or 2-part models were not attempted to avoid overfitting. Each model was first estimated using DLQI alone (Level 1, without covariates) and then as an extended model (Level 2, including age, sex and self-rated disease severity as covariates).

Further details on model specification, selection rationale and the comparative evaluation of model properties are provided in the Appendix S2 (Table S1).

### Prediction and model performance

Predicted EQ-5D5-5L utilities were generated for each model, following MAPS (17) recommendations to ensure reproducibility. For comparability, all predictions were restricted to the Swedish value set (−0.314 to 1.0). For OLS regression, values below −0.314 or above 1.0 were truncated to these bounds.

Model performance was evaluated using the root mean squared error (RMSE) and the mean absolute error (MAE). These prediction error metrics were the primary basis for model selection. Lower RMSE and MAE indicated better predictive accuracy. In addition, the squared Pearson correlation (*R* ^2^) between observed and predicted utilities for all models was reported, as a descriptive measure of predictive fit.

Coefficients, standard errors and confidence intervals for the best-performing model were estimated and are reported in the Results, while the variance–covariance matrix is provided in the supplementary material.

### Model diagnostics and validation

Residual distributions, influence statistics and calibration plots were examined to assess model adequacy and boundary behaviour. Internal validation was performed using bootstrap resampling and repeated *k*-fold cross-validation to evaluate model stability and generalizability.

All analyses were conducted in Stata version 18 (StataCorp LLC, College Station, TX, USA).

## RESULTS

### Study population

A total of 112 adults with physician-diagnosed AD were included. The median age was 46.0 years (interquartile range: IQR=23) and 87.5% were women. Self-rated disease severity was severe in 8.9%, moderate in 21.4%, mild in 33.9% and clear or almost clear in 35.7%. Comorbid allergic conditions were common, including food allergy (64%), asthma (54%) and allergic rhinitis (47%). In addition, 23% reported anxiety and 14% depression.

**Fig. 1.**
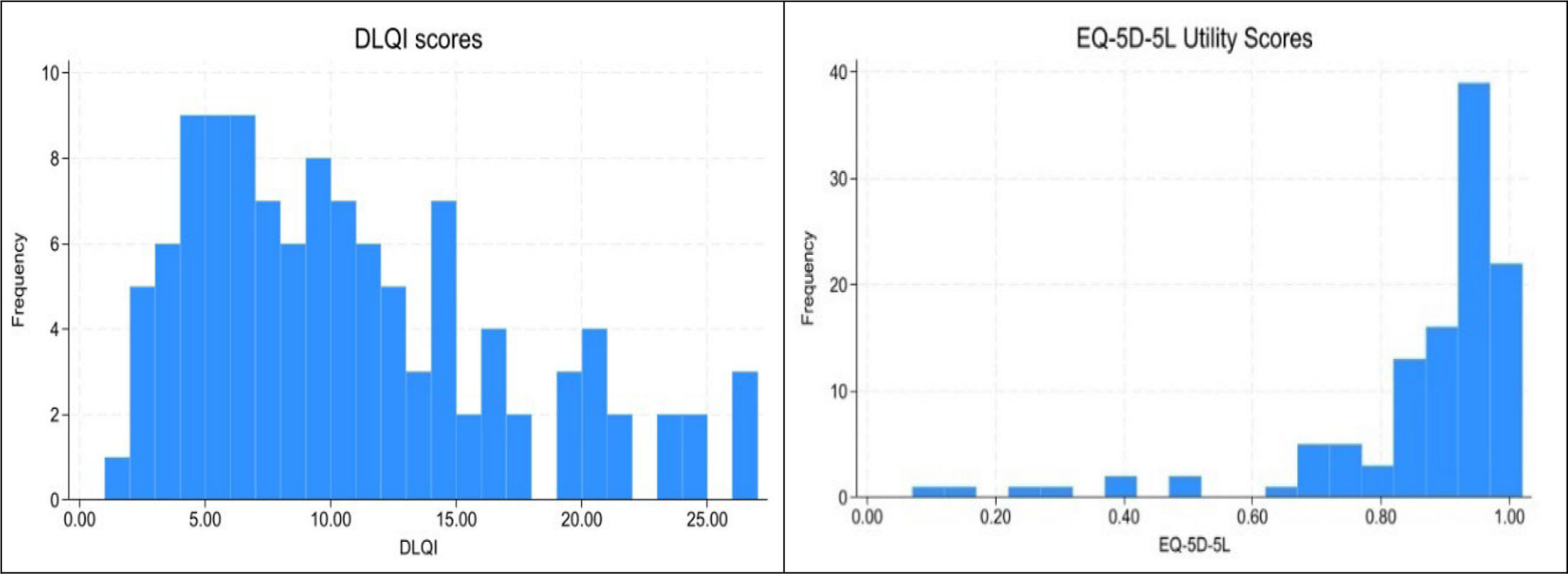
Distributions of the Dermatology Life Quality Index (DLQI) and the EuroQol Five Dimensions Five Levels (EQ-5D-5L) health index (utility) scores.

**Fig. 2.**
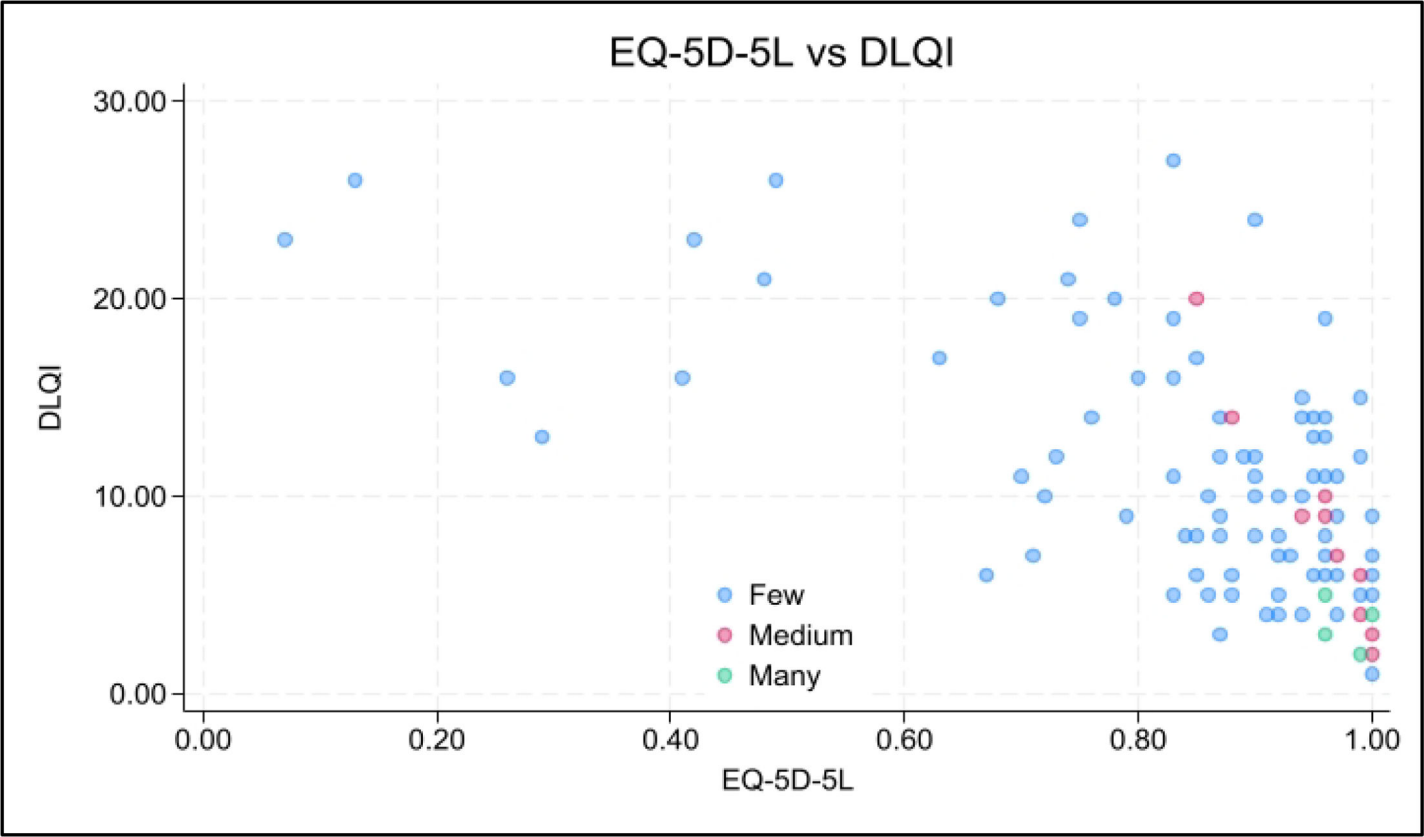
Scatterplot of the Dermatology Life Quality Index (DLQI) and the EuroQol Five Dimensions Five Levels (EQ-5D-5L) health index score with point colour by frequency. Point colours represent local data density (blue = few, pink = moderate, green = many overlapping observations). The plot shows the expected negative association between worsening DLQI (higher values) and lower EQ-5D-5L utility.

Mean DLQI was 10.2 (SD 6.3), indicating a moderate impact on daily life (Table I). The mean EQ-5D-5L index was 0.86 (SD 0.18) and mean EQ-VAS 64.2 (SD 20.6). EQ-5D-5L values showed a ceiling effect (10.7% at 1.0). Distributions and the inverse DLQI-EQ-5D-5L relationship are shown in Figs 1 and 2.

### Conceptual overlap

The DLQI total scores showed a strong inverse correlation with EQ-5D-5L utilities (*r*=−0.64, *p*<0.001), demonstrating good conceptual overlap between dermatology-specific and generic HRQoL. A detailed theoretical mapping of item-level relationships between the two instruments is provided in the Appendix S3 (Table S2).

**Fig. 3.**
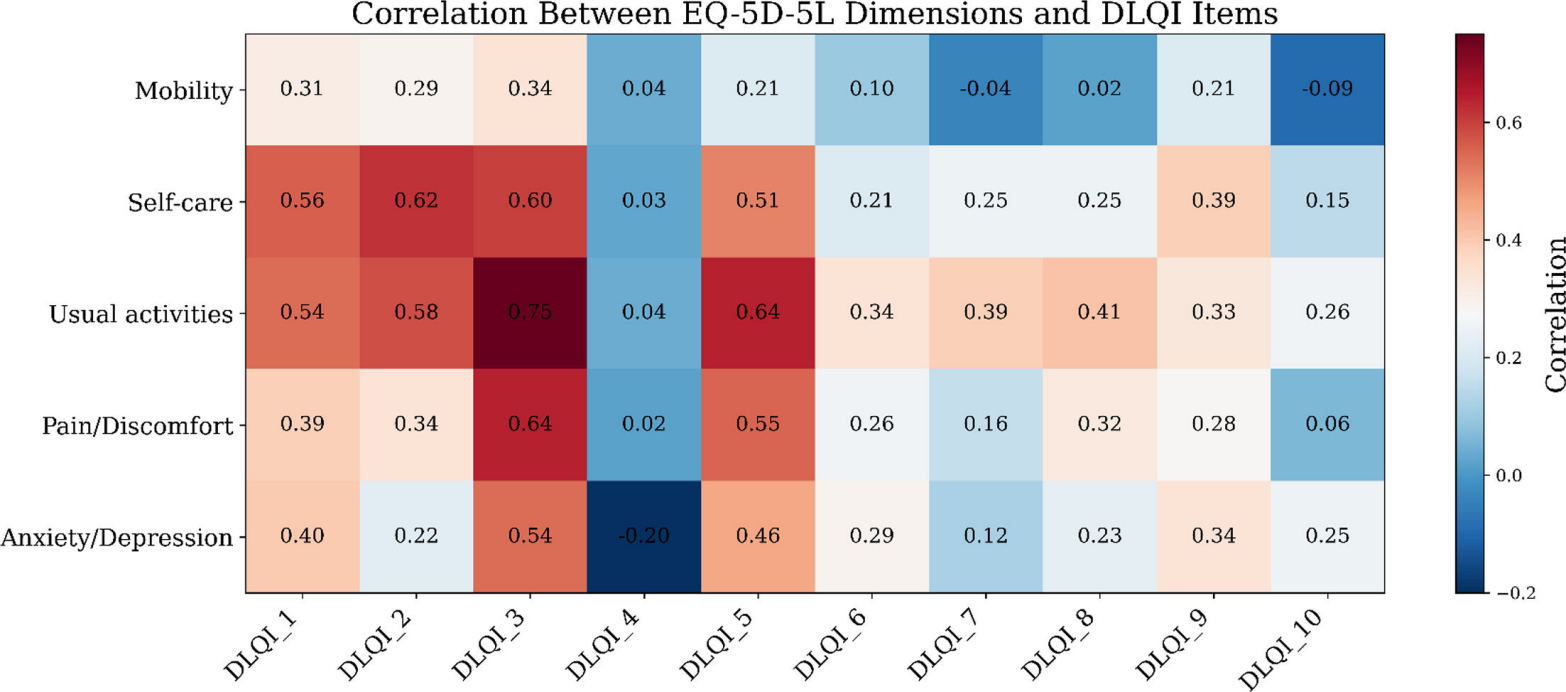
Conceptual overlap of the Dermatology Life Quality Index (DLQI) domains with the EQ-5D-5L=EuroQol 5 Dimensions 5 Levels (EQ-5D-5L) dimensions in the study. Spearman correlation coefficients between DLQI items and EQ-5D-5L dimensions. Colour intensity reflects the strength and direction of the correlation (darker red shades=stronger positive correlations, darker blue=stronger negative correlations, lighter shades=weaker correlations). More details on the theoretical relationship/overlap of DLQI domains to EQ-5D-5L dimensions are provided in the .Explanations of the domains for each instrument: EQ-5D-5L dimensions: 1.Mobility, 2. Self-care, 3. Usual activities, 4. Pain/discomfort, 5. Anxiety/depression. DLQI domains and corresponding items (Q1-Q10): Symptoms and Feelings: Q1,Q2, Daily Activities: Q3-Q4, Leisure: Q5-Q6, Work and School: Q7, Personal Relationships: Q8-Q9, Treatment: Q10.

The strongest item-level correlations occurred between DLQI daily-activity and leisure items and the EQ-5D-5L “usual activities” and “pain/discomfort” dimensions (*r*≈0.6–0.7), while mobility correlated weakly (Fig. 3). These findings confirmed good alignment between DLQI and EQ-5D-5L constructs and provided a rationale for developing a statistical mapping model to predict EQ-5D-5L utilities.

### Model performance and comparison

**Fig. 4.**
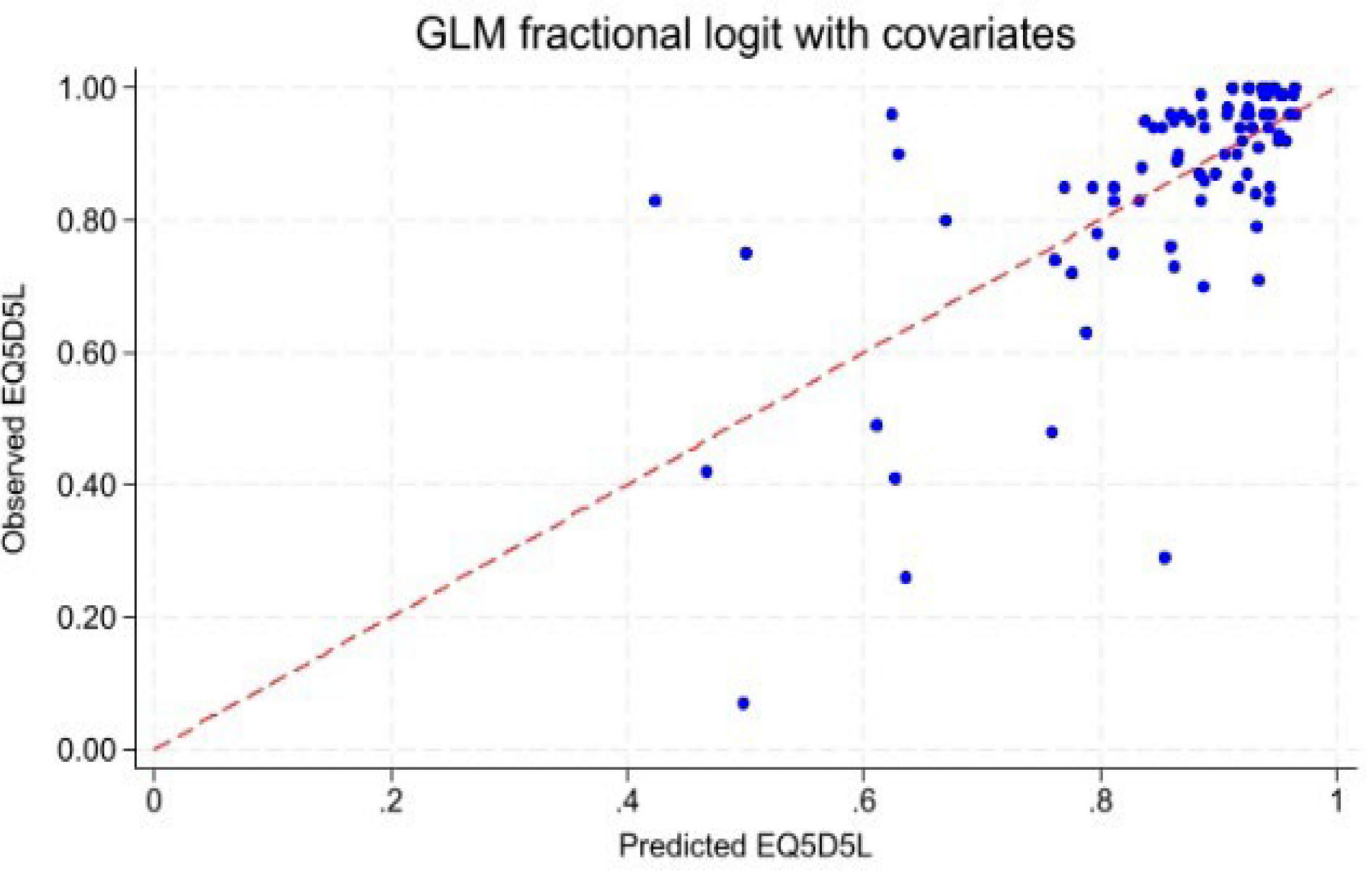
Observed vs predicted values from the best-performing model (GLM-fl with covariates). The plot compares observed and predicted EQ-5D-5L values from the best-performing fractional logit (GLM-fl) model including age, sex, severity. Points lie close to the diagonal line, indicating good agreement with slight over-prediction at lower utility values. DLQI: Dermatology Life Quality Index; EQ-5D-5L: EuroQol 5 Dimensions 5 Levels; GLM-fl: Generalized Linear Model- fractional logit.

Table II summarizes performance statistics for each model. Predicted mean EQ-5D-5L values were close to the observed mean (0.865), showing minimal bias. Among level-1 models (DLQI only, without covariates), Tobit achieved the lowest RMSE (0.141), while GLM-fl had the lowest MAE (0.089). Adding age, sex and severity as covariates (Level 2: extended models) improved accuracy across all models; GLM-fl and OLS had the lowest MAE (0.088±0.001). Considering predictive accuracy, boundedness and interpretability, the fractional logit model with covariates was selected as the final preferred model. Fig. 4 graphically presents the relationship between the observed and predicted EQ-5D-5L values for the best performing model, the GLM-fl, demonstrating close calibration with covariates.

**Table II.** Performance metrics for each model and level

	Mean overall EQ-5D-5L observed	SE				
	0.8652	0.01689	Models’ performance			
Models	Mean overall EQ-5D-5L predicted	SE	RMSE	MAE	(corr) *R* ^2^	Ranking
Level 1						
OLS	0.8625	0.0126	0.1427	0.0937	0.368 (adj. *R* ^2^=0.362)	6
Tobit	0.8468	0.0345	0.1414	0.0979	0.384	7
OIBR	0.8499	0.0084	0.1432	0.0978	0.380	8
GLM-fl	0.8624	0.0109	0.1397	0.0893	0.384	5
Level 2						
OLS	0.8605	0.0126	0.1395	0.0884	0.362 (adj. *R* ^2^=0.363)	3
Tobit	0.8450	0.0348	0.1344	0.0921	0.359	2
OIBR	0.8477	0.0097	0.1365	0.0927	0.394	4
GLM-fl	0.8590	0.0146	0.1351	0.0877	0.393	1

RMSE and MAE are the primary indicators of predictive accuracy. The column labelled “(corr) *R*²” refers to the squared Pearson correlation coefficient between observed and predicted EQ-5D-5L values and is included as a descriptive measure of model fit. It is not equivalent to the coefficient of determination (R²) or adjusted *R*² used in linear regression.

adj.:adjusted; DLQI:Dermatology Life Quality Index; EQ-5D-5L:EuroQol 5 Dimensions 5 Levels; GLM-fl:Generalized Linear Model with a fractional logit link; Level 1:no covariates; Level 2:extended model incl. age, sex, and severity as covariates; OIBR:One-Inflated Beta Regression; OLS:Ordinary Least Squares regression; SE:Standard Error; TOBIT:Tobit regression model.

All scatterplots of the observed vs predicted EQ-5D-5L utility values for all models investigated are presented in the Appendix S4.

### Best model coefficients and prediction algorithm

Coefficient estimates for the preferred model are shown in Table III. DLQI was the only statistically significant predictor of EQ-5D-5L utility values (β=−0.106 ± 0.024, *p*<0.001). A 5-point increase in DLQI corresponded on average to a 0.07 reduction in EQ-5D-5L utility.

**Table III.** Coefficient statistics for the best-performing model (GLM-fl with covariates)

Variable	Coefficient	SE	*p*-value	95% CI
DLQI	−0.1061	0.0235	0.000	−0.15–-0.060
Age	−0.0096	0.0089	0.280	−0.027–0.008
Sex (male)	0.1303	0.3176	0.682	−0.492–0.752
Severity (severe)	−0.7518	0.5966	0.208	−1.921–0.417
Severity (moderate)	0.0544	0.4471	0.903	−0.822–0.931
Severity (mild)	0.2323	0.3934	0.555	−0.538–1.003
Constant	3.6069	0.5242	0.000	2.579–4.633

DLQI is continuous (higher = worse). Coefficients are on the logit scale.

CI:confidence interval; DLQI:Dermatology Life Quality Index; GLM-fl:Generalized Linear Model with a fractional logit link with covariates age, sex, severity; SE:standard error; severity reference:almost/no problem; sex reference:female.

The expected EQ-5D-5L score for individual i can be predicted using the equation.

EQ-5D-5L_i_=exp(η_i_)/(1+exp(η_i_)), with the linear predictor defined as.

η_i_=3.607 − (0.1061·DLQI_i_) − (0.0096·age_i_) + (0.1303·sex_i_) + (0.2323·severity mild_i_) + (0.0544·severity moderate_i_) − (0.7518·severity severe_i_), where sex=1 for men (0 for women), severity dummies equal 1 if the person is in that category and 0 otherwise.

### Model diagnostics and validation

**Fig. 5.**
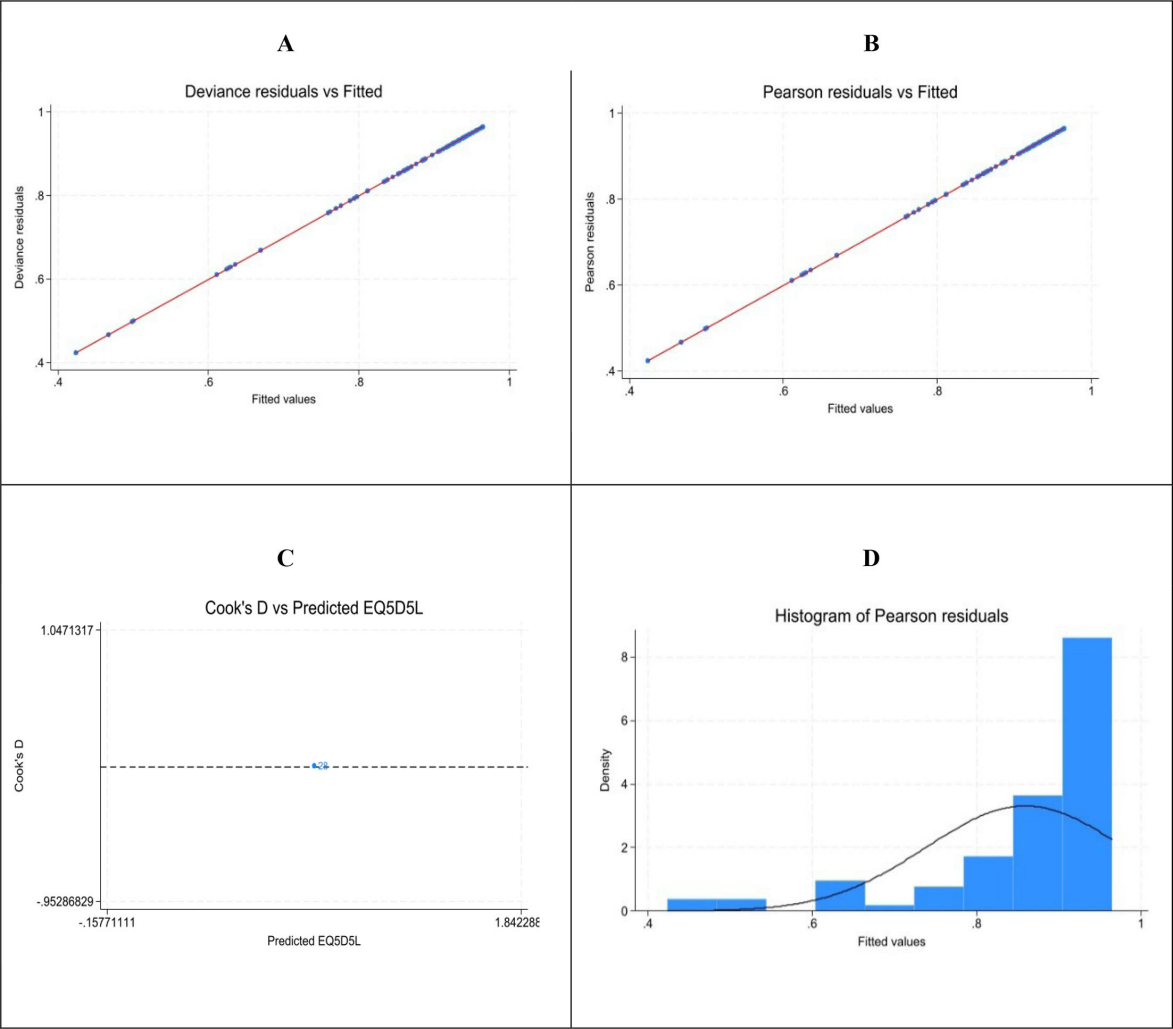
Residual analysis of the GLM-fl model with covariates. A. Deviance residuals vs fitted values, B. Pearson residuals vs fitted values, C. Cook distance vs fitted values, D. Histogram of Pearson residuals. Abbreviations: Cook D = Cook’s distance, EQ-5D-5L = EuroQol Five Dimensions Five Levels, GLM-fl=Generalized Linear Model-fractional logit, including covariates age, sex, severity. Note: Residuals are centred around 0, show mild skewness, and exhibit no influential observations.

Model diagnostics confirmed that residuals were centred around 0 with no influential outliers (Fig. 5). A mild positive skew was observed at higher residual values, which reflects the typical right-skewed and ceiling-effect pattern of EQ-5D-5L data rather than model misfit.

Cross-validation and bootstrap resampling supported stable predictive performance, in line with the ISPOR validation criteria (19). In repeated 10-fold cross-validation, the model showed consistent accuracy (MAE=0.075, SD 0.002; RMSE=0.119, SD 0.005) and low variability across folds, indicating that results were not driven by any single subgroup of participants. The model also explained 27% of the variance in EQ-5D-5L values, indicating moderate predictive performance. Bootstrap validation (100 replications) produced similar apparent and optimism-corrected errors (MAE≈0.09–0.10), suggesting limited overfitting. This indicates that the model’s performance was stable and is likely to be reliable beyond the original sample. The correlation between observed and predicted EQ-5D-5L utility values was 0.65, demonstrating good overall calibration.

Additional details on the residual and validation analyses are provided in the Appendices S5-S7 including the variance-covariance matrix of the estimated coefficients (Appendix S8).

## DISCUSSION

### Main findings

This study demonstrated a clear and conceptually meaningful association between dermatology-specific quality of life (DLQI) and generic health status (EQ-5D-5L) in adults with AD. The strongest overlap occurred in domains related to daily functioning, leisure and symptom burden, confirming that DLQI captures much of the same construct as EQ-5D-5L. This indicates that DLQI can serve as a meaningful proxy for patients” general health perceptions, when preference-based measures are unavailable. A ceiling effect in EQ-5D-5L values suggested reduced sensitivity to mild impairments implying that the burden of AD may be underestimated when only generic measures are used, as noted in prior studies of dermatological populations (27–29). Conversely, disease-specific instruments such as the DLQI may exhibit differences in burden estimates when compared with generic measures by emphasizing dermatology-related aspects of quality of life (30–32).

Among the tested models, it should be noted that the performance differences were small, and the fractional logit generalized linear model with covariates provided the best balance of predictive accuracy and interpretability. Internal validation supported stable performance and good calibration, although predictive strength was moderate, consistent with previous mapping studies of DLQI and EQ-5D (10, 11, 30, 33, 34). These findings show that DLQI can be used to estimate EQ-5D-5L utilities when direct preference-based data are not available.

### Methodological considerations

The EQ-5D-5L utility scale often clusters at full health, leading to ceiling effects (35). This pattern reduces variability in utility values and therefore limits the ability of mapping models to detect differences in health status (36). Fractional logit regression is appropriate for such data, as it constrains predictions within the valid range and accounts for asymmetry near the boundaries (23, 24). Although beta and inflated-beta models can model boundary inflation, they generally require larger samples for stable estimation (26, 36, 37). Given the modest dataset, we prioritized established parsimonious models, such as ordinary least squares, Tobit, fractional logit and one-inflated beta regression, in line with recognized mapping guidelines and prior literature (17–19, 38, 39).

Total DLQI scores were used instead of item-level data to ensure model stability and avoid overfitting. While item-level mapping can improve precision, it increases complexity and parameter demands, particularly in smaller datasets (31, 33, 36). Including age, sex and self-rated disease severity improved model performance, consistent with earlier mapping studies in AD and other skin conditions (10, 11, 30). The chosen fractional logit model thus provided a sound compromise between theoretical suitability, practical robustness and interpretability.

### Comparison with previous research

The present findings align closely with previous DLQI-EQ-5D mapping studies. Ali et al. (10) analysed over 3,500 patients with various skin diseases across Europe, demonstrating that DLQI could predict EQ-5D-3L utilities using an ordinal logistic regression model with small mean errors and strong inverse correlations. Vilsbøll et al. (11) focused specifically on AD, analysing more than 1,200 patients from Europe and the USA. Their best-performing mixture regression model, which incorporated DLQI along with age and sex, achieved mean absolute and RMSEs of 0.079 and 0.113, respectively, with a correlation of approximately −0.51. Similar performance metrics were reported mapping studies on psoriasis (30, 33, 34), which also found moderate predictive accuracy using DLQI as the primary predictor.

Compared with these studies, our model was based on a smaller, Swedish-specific sample but applied comparable analytic principles. The observed correlation (*r*=−0.64) and predictive precision (MAE=0.075) were similar to those reported in international datasets (10, 11, 31, 32), where correlations typically range from approximately −0.5 to −0.7 and MAE values from 0.07 to 0.10, supporting the robustness of the DLQI-EQ-5D relationship observed in this study.

### Strengths and limitations

This is, to our knowledge, the first study to estimate EQ-5D-5L utilities from DLQI scores in a Swedish AD population. Unlike earlier models that used international or EQ-5D-3L value sets, our study employed the Swedish EQ-5D-5L valuation, providing locally relevant utility estimates for use in national health economic evaluations and clinical registries. The analysis followed established methodological guidance (17–19, 38, 39), compared multiple model specifications and included comprehensive diagnostics and internal validation to strengthen robustness.

Several limitations should be acknowledged. The sample was modest and predominantly female, which may limit generalizability. However, this distribution broadly reflects the epidemiology of adult AD, where women often have higher prevalence and greater quality-of-life burden (1–4), although the magnitude observed in this sample was higher. Thus, while not representative of all adults with AD, the sample captures the subgroup most affected by disease impact. The study relied on self-reported data, and external validation was not possible. Residual skewness and non-normality were consistent with bounded utility data and small-sample characteristics (23, 25), indicating moderate rather than high predictive precision. The model should therefore be regarded as exploratory and suitable for population-level rather than individual predictions. Future research using larger, clinically verified datasets is needed to refine and externally validate the algorithm. In addition, participants were recruited through the Swedish Asthma and Allergy Patient Association, which may limit the representativeness of the general adult AD population, as members of patient organizations may differ from nonmembers in terms of greater disease burden, higher engagement with healthcare and increased reporting of symptoms.

### Significance and practical relevance

This exploratory study provides the first Swedish-specific link between DLQI and EQ-5D-5L in adults with AD. The results show that disease-specific impairments correspond to measurable reductions in general health, helping healthcare professionals conceptually understand DLQI scores in a broader health context. This offers clearer insight into how changes in DLQI may relate to overall HRQoL and may support communication with patients and decision-makers.

At a population level, the mapping model enables DLQI data, commonly collected in clinical studies and registries, to be used for estimating EQ-5D-5L utilities. Importantly, the DLQI is recommended by the Harmonising Outcome Measures for Eczema (HOME) (8) initiative as a core patient-reported outcome for AD clinical trials and is routinely collected in the Swedish national AD registry (SwedAD) (40). By providing a method for deriving utilities when preference-based data are unavailable, this study facilitates the use of existing DLQI datasets in health economic analyses, including cost-effectiveness and health-technology assessments (17, 18). Although not intended for individual prediction, the present mapping approach offers a practical foundation for integrating dermatology-specific outcomes into Swedish health economic evaluations and policy decision-making.

### Conclusions

In summary, this exploratory study developed the first Swedish DLQI-EQ-5D-5L mapping model for adults with AD. The fractional logit model, including age, sex and severity level as covariates, offered the best combination of fit and predictive accuracy. Although performance was moderate, DLQI scores meaningfully reflected generic health status. This work provides a useful basis for integrating dermatology-specific outcomes into health economic research, for informing broader patient assessments and for supporting healthcare policy frameworks.

## Data Availability

The data that support the findings of this study are available from the corresponding author upon reasonable request.
