# Supplementary material for: Relationship between Dermatology Life Quality Index Scores and EQ-5D-5L Utility Values in Adults with Atopic Dermatitis: Development of a Swedish Mapping Model
Source: Acta Derm Venereol. 2026 Jun 2;106:0277. doi: 10.2340/actadv.v106.adv-2025-0277 (PMC13230340; doi:10.2340/actadv.v106.adv-2025-0277)
Supplement: Supplementary Material 1. [file ActaDv-106-0277-s0001.pdf]

Supplementary material has been published as submitted. It has not been copyedited, or typeset by Acta Dermato-Venereologica

APPENDIX S1. Recruitment and survey procedure

Study design and population

A cross-sectional, online survey was conducted from January 2024 to January 2025 among adult members of the Swedish Asthma and Allergy Patient Association. This 12-month period was chosen to capture potential seasonal variation in AD activity and related costs; respondents completed the questionnaire once, reporting for the previous month.

Inclusion criteria were: (i) age ≥ 18 years, (ii) residence in Sweden, (iii) self-reported current or previous AD. Exclusion criteria were incomplete consent or missing key demographic or AD-related data. AD status and severity were self-reported.

Primary variables were AD severity, HRQoL, work productivity (absenteeism, presenteeism and total productivity loss), activity impairment, out-of-pocket costs, willingness-to-pay (WTP) for symptom relief, perceived treatment benefit, and healthcare utilisation.

Survey development and data collection

The questionnaire was designed collaboratively by physicians, health economics researchers and patients using the Delphi process. Clinical experts ensured content validity, methodologists refined question structure and recall windows, and patients reviewed relevance and wording.

The survey collected data on:

- demographics (age, sex)
- age at AD diagnosis
- self-rated AD severity during the past month
    - The self-reported AD severity was assessed using the Patient Global Assessment (PGA), a measure supported by prior literature, including validation studies in adults with AD*.

      *** Example regarding validation:**
      Vakharia PP, Chopra R, Sacotte R, Patel N, Immaneni S, White T, Kantor R, Hsu DY, Silverberg JI. Validation of patient-reported global severity of atopic dermatitis in adults. Allergy. 2018 Feb;73(2):451-458. doi: 10.1111/all.13309.

      Example/supporting use in adult AD research and further evidence for the content and construct validity:
      Silverberg JI, Chiesa Fuxench ZC, Gelfand JM, Margolis DJ, Boguniewicz M, Fonacier L, Grayson MH, Simpson EL, Ong PY. Content and construct validity, predictors, and distribution of self-reported atopic dermatitis severity in US adults. Ann Allergy Asthma Immunol. 2018 Dec;121(6):729-734.e4. doi: 10.1016/j.anai.2018.07.040.
- atopic comorbidities (asthma, allergic rhinitis, food allergy) and non-atopic comorbidities (depression, anxiety, ADHD, eating disorders, other chronic conditions)
- HRQoL
- work productivity and reasons for absenteeism (for example, physician visits)
- daily activity limitations
- healthcare contacts and treatments used
- perceived treatment benefit
- monthly out-of-pocket expenses for treatment, travel, and skincare products

Procedure

The survey was distributed by email through the Patient Association. Data were collected via the SurveyMonkey® platform. Participants provided electronic informed consent before accessing the questionnaire and could contact the research team for clarification. Each record represented one individual's experience over a one-month recall period. Responses were pseudonymised before analysis. Each participant could respond once during the year. Participation was voluntary and non-remunerated.

APPENDIX S2. Overview of regression models used for EQ-5D mapping

Table S1. Summary of common model types for mapping analysis

Model Type	Sub-Type / Example	When to Use	Key Characteristics	Advantages	Limitations
**Linear Models**	OLS (Ordinary Least Squares)	When data is approximately linear, utility scores roughly normal	Simple linear regression	Easy to implement and interpret	Poor with skewed/bounded data
	GLM (Generalised Linear Models)	For skewed, bounded, or heteroscedastic data	Allows non-normal distribution (e.g., gamma, log link)	More flexible than OLS	Requires careful choice of family/link
	Tobit	When data is censored (e.g., utility scores bounded at 1)	Accounts for upper/lower bounds in dependent variable	Handles censored utility values well	Assumes normality and homoscedasticity
	CLAD (Censored Least Absolute Deviations)	Robust to outliers and censored data	Minimizes absolute error, not squared error	Resistant to heteroscedasticity	Less commonly used, more complex
**Two-Part Models**	Logit + OLS/GLM	When many observations are at full health (1.0)	First part predicts probability of full health, second predicts value <1	Handles "spike at full health"	Requires large sample to fit both parts reliably
**Ordinal Models**	Ordinal Logistic/Probit Regression	When mapping to domain-level utility states (e.g., EQ-5D responses)	Models ordered outcomes rather than utility directly	Useful for response mapping	Must simulate final utility using predicted responses
**Response Mapping**	Multivariate Ordered Logit or Multinomial Logit	Predicts levels of individual domains (not utility directly)	Indirect approach via predicted domain profiles	Closer to actual EQ-5D structure	Requires post-processing to calculate utility
**Mixture Models**	Regression Mixture Models	When different subgroups show distinct patterns	Mixture of distributions (e.g., high vs. low utility)	Captures unobserved heterogeneity	Complex to interpret; prone to overfitting
**Beta Regression**	Beta or Zero-One Inflated Beta (ZOIB)	For data bounded between 0 and 1	Models continuous data in (0,1); ZOIB handles spikes at 0 or 1	Fits skewed, bounded data well	Can't handle exact 0 or 1 values without inflation
**ALDVMM**	Adjusted Limited Dependent Variable Mixture Model	Specialized for mapping EQ-5D	Mixture model that accounts for bounded, multimodal utility data	High predictive accuracy for EQ-5D	Complex to estimate and interpret
**Machine Learning**	Random Forest, XGBoost, Neural Nets	When data is large and non-linear relationships are expected	Data-driven, non-parametric	Can capture complex interactions	Often less interpretable, overfitting risk

APPENDIX S3. Conceptual overlap between DLQI and EQ-5D-5L

Table S2. Theoretical overlap of DLQI domains with EQ-5D-5L

DLQI domain	EQ-5D-5L overlap	Comment
Symptoms and Feelings	Pain/Discomfort, Anxiety/Depression	Physical & psychological symptoms are shared.
Daily Activities	Usual Activities	Interference in daily function overlaps.
Leisure	Usual Activities, Anxiety/Depression	Affects enjoyment and participation.
Work and School	Usual Activities	Functional capacity for work overlaps.
Personal Relationships	Anxiety/Depression	Psychological well-being impacts relationships.
Treatment	Not applicable	Not directly represented in EQ-5D-5L.

APPENDIX S4. Observed vs predicted values across - all models

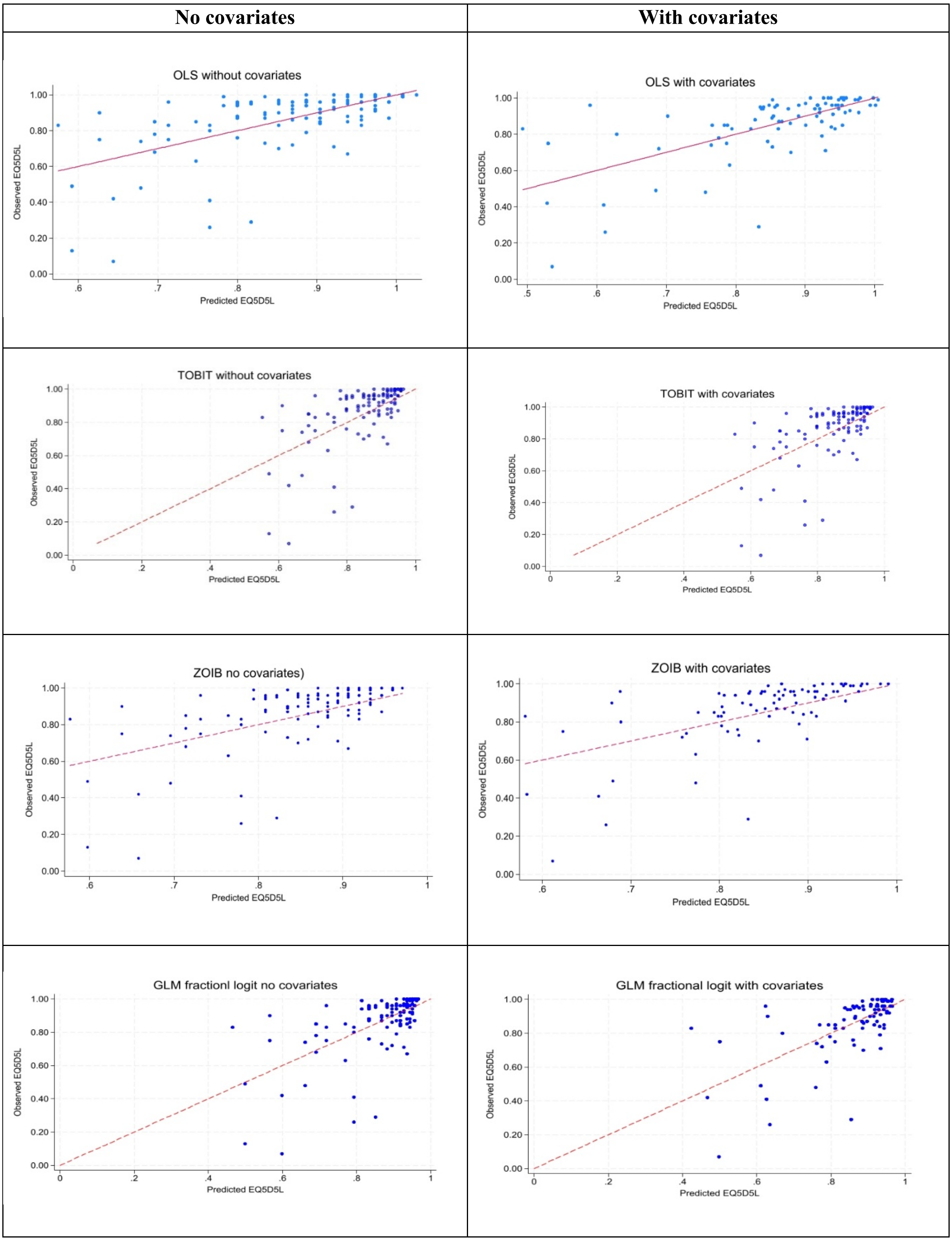

Fig. S1. Observed vs predicted values across all models with covariates and without covariates

Abbreviations: DLQI=dermatology life quality index, EQ-5D-5L=-EuroQol 5 Dimensions OLS=Ordinary Least Squares, GLM= Generalised Linear Model, ZOIB-=(Zero) One-Inflated--Beta regression

APPENDIX S5. Residual analysis (extra graphs, summary, code)

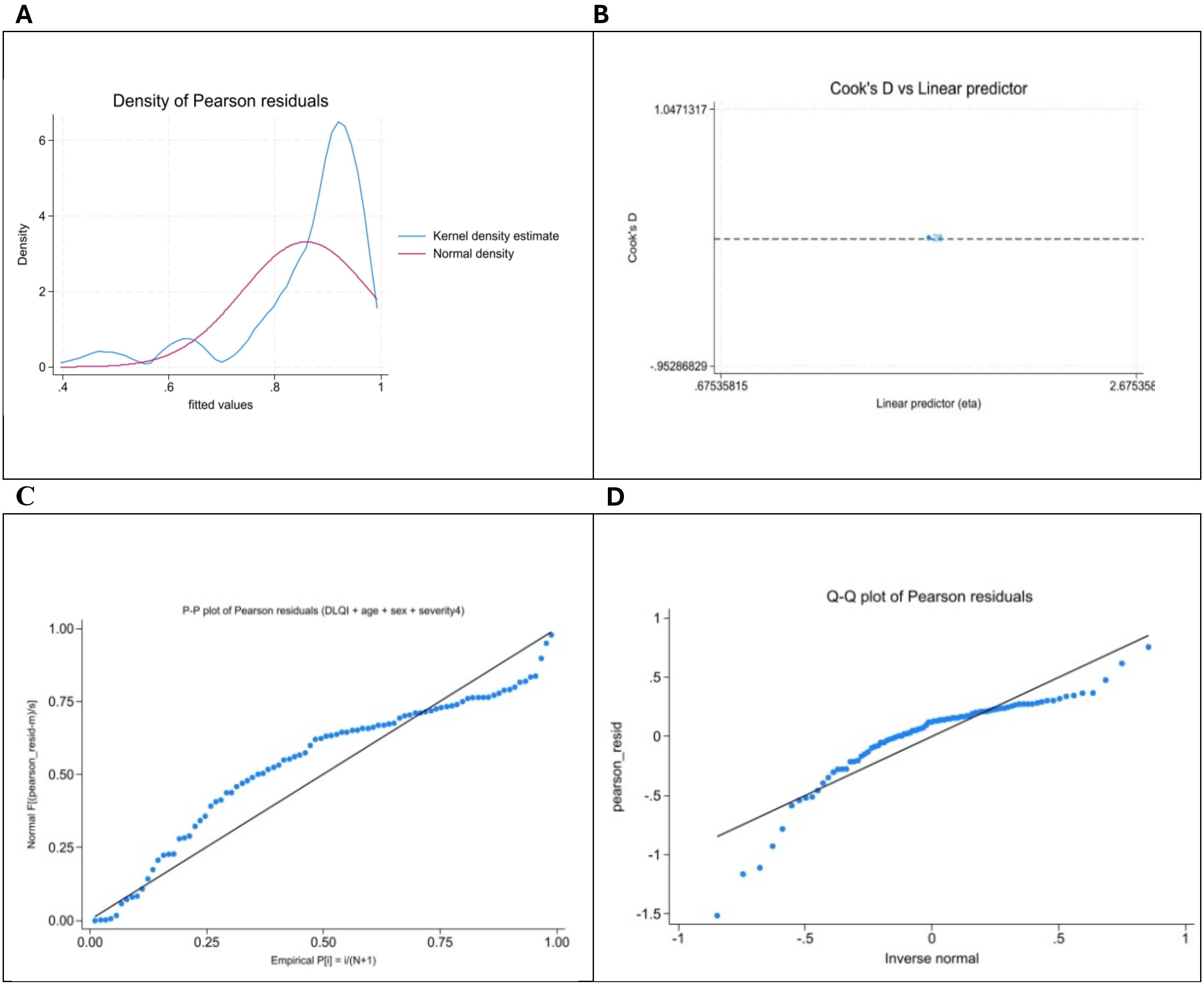

Fig. S2. A Deviance residuals vs. fitted values, B. Pearson residuals vs. fitted values, C. Kernel density of Pearson residuals, D. Q-Q plot of Pearson residuals

Notes: A: scatterplot of deviance residuals against fitted EQ-5D-5L values with a linear fit line. Residuals are centred around zero, with no major outliers, suggesting no strong violation of model assumptions, **B**: Pearson residuals plotted against fitted EQ-5D-5L values. Skewness and increasing spread at higher fitted values indicate some model misfit but no extreme outliers, **C:** Kernel density estimate with normal reference line. Deviations from normality are visible, particularly in the upper tail, **D:** Normal Q-Q plot showing departures from the 45° line at the upper quantiles, indicating skewness

Summary-comments

- Deviance and Pearson residuals were centred around zero, with no evidence of highly influential outliers (based on Cook's distance).
- Pearson residuals showed skewness and deviations from normality, especially at higher fitted values**.**
- Overall patterns indicate a reasonable model fit, with some moderate mis-specification, consistent with the model's predictive performance in validation.

Code (key steps-whole code can be provided upon request)

```
* Fit fractional logit model
fracreg logit EQ5D5L DLQI age sex i.severity4

* Fitted values and residuals
predict YHAT, mu
predict R_DEV, dev
```

```
predict R_PEARSON, pr

* Residual-fitted plots
twoway (scatter R_DEV YHAT) (lfit R_DEV YHAT),
    title("Deviance residuals vs fitted") yline(0)
twoway (scatter R_PEARSON YHAT) (lfit R_PEARSON YHAT),
    title("Pearson residuals vs fitted") yline(0)

* Distribution of residuals
histogram R_PEARSON, normal
kdensity R_PEARSON, normal
```

APPENDIX S6. Internal validation: bootstrap

(Results, Interpretation & Code)

Key results

Metric	Apparent	Optimism-corrected
R^2	0.424	0.318
RMSE	0.135	0.156
MAE	0.088	0.098

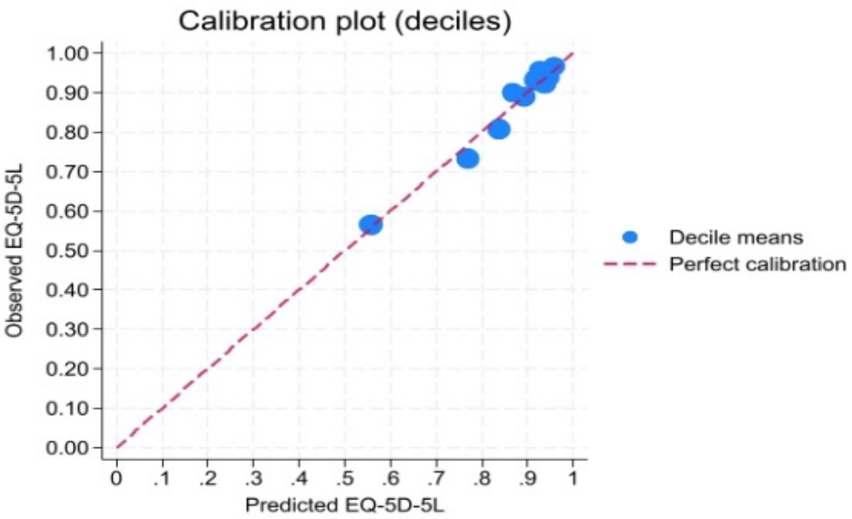

Fig. S3. Internal validation - Calibration plot for the fractional logit model predicting EQ-5D-5L from DLQI, age, sex, and severity.

Note: Observed mean EQ-5D-5L values (y-axis) are plotted against predicted values (x-axis) across deciles of predicted risk. The dashed diagonal line represents perfect calibration. The model shows good calibration across most of the prediction range, with slight over-prediction at lower predicted values.

Interpretation

- After optimism correction, the model explained ~32% of the variance in EQ-5D-5L, consistent with moderate mapping performance.
- Prediction error was small: MAE ≈ 0.10, indicating differences of about one-tenth of a utility unit on average.
- Optimism was modest (≈0.10 in R^2 and ≈0.02 in error metrics), suggesting limited overfitting despite a relatively small sample.
- Calibration was good overall: the calibration curve showed close agreement between observed and predicted values across most deciles, with slight over-prediction at lower EQ-5D-5L values.

Conclusions

The bootstrap indicates that the fractional logit model (DLQI + age + sex + severity) has moderate explanatory power, small prediction errors, and limited optimism bias. Calibration is generally good. The model appears suitable for predicting EQ-5D-5L from DLQI in similar populations, although external validation in larger datasets is recommended.

Code

```
* Fit model on bootstrap sample
    fracreg logit EQ5D5L DLQI age i.sex i.severity4
    predict xb, xb
    gen ph = invlogit(xb)
    corr EQ5D5L ph
    local R2_app = r(rho)^2
    * Apply model to original dataset
    preserve
```

```
        use "$ORIGFILE", clear
        estimates restore bootfit
        predict xb2, xb
        gen ph2 = invlogit(xb2)
        corr EQ5D5L ph2
        local R2_test = r(rho)^2
    restore
    * Return results
    return scalar R2_app = `R2_app'
    return scalar R2_test = `R2_test'
    return scalar optimism = `R2_app' - `R2_test'
end
```

APPENDIX S7. Internal validation by repeated 10-fold cross-validation (Results, Interpretation & Code)

Mean performance across repeats

Metric	Mean	SD	Interpretation
**MAE**	**0.075**	**0.002**	**Small error; stable**
**RMSE**	**0.119**	**0.005**	**Low to moderate**
**R^2**	**0.272**	**0.034**	**Moderate predictive power**
**Calibration-in-the-large**	**0.0015**	**0.0017**	**No systematic bias**

- **MAE:** On average, predictions are off by ~0.08 on the [0, 1] EQ-5D scale. Very consistent across repeats.
- **RMSE:** A bit more sensitive to outliers, but again stable.
- **R^2:** The model explains about 27% of the variance in EQ-5D01.
- **Calibration-in-the-large:** Mean predicted ≈ mean observed. No systematic over- or under-prediction.

Interpretation

- **Accuracy:** Errors are small relative to EQ-5D's range. MAE ~0.08 is reasonable for a mapping model.
- **Explained variance:** 27% R^2 is modest but in line with other similar mapping studies.
- **Stability:** Small SDs (MAE ±0.002, RMSE ±0.005) show your results are robust to fold assignment; Performance is stable and consistent across repeats.
- **Calibration:** Essentially unbiased (mean predicted ≈ mean observed), predictions don't systematically overshoot or undershoot.

Code (Key steps, whole code can be provided upon request)

```
* 1. Scale EQ-5D to 0–1
gen EQ5D01 = (EQ5D5L + 0.314)/1.314
replace EQ5D01 = min(max(EQ5D01,0),1)
* 2. Set number of repetitions and folds
local REPS 50
local K 10
* 3. Loop over repetitions:
forvalues r = 1/`REPS' {
   - Randomize data order
   - Assign observations to K folds
   - For each fold:
       Fit GLM on training folds
       Predict on held-out fold
   - Compute MAE, RMSE, R²
   - Store results
* 4. Save summary statistics
```

APPENDIX S8. Variance - Covariance Matrix (Results, Interpretation & Code)

Purpose

The variance-covariance matrix provides the variances (diagonal) and covariances (off-diagonal) of the estimated regression coefficients from the fractional logit model. It is used for:

- estimating standard errors ,
- hypothesis tests and confidence intervals,
- assessing relationships among coefficients, and
- identifying potential multicollinearity.
- further uncertainty analysis and for simulations relevant in health economics (e.g. probabilistic sensitivity analysis (PSA), uncertainty in QALY estimation)

Variance-covariance matrix of the fractional logit model

symmetric V[9,9]

	Y01: DLQI	Y01: age	Y01: 1b. sex	Y01: 2. sex	Y01: 1b. severity4	Y01: 2. severity4	Y01: 3. severity4	Y01: 4. severity4	Y01: _cons
Y01:DLQI	.00058134								
Y01:age	-.00001235	.0000826							
Y01:1b.sex	0	0	0						
Y01:2.sex	-.0001528	-.00075304	0	.10478186					
Y01:1b.severity4	0	0	0	0	0				
Y01:2.severity4	-.00556424	.00077148	0	.00161786	0	.15845181			
Y01:3.severity4	-.00623198	.00091749	0	.00003374	0	.15107192	.20494004		
Y01:4.severity4	-.00753674	-.00051691	0	-.01574669	0	.15492105	.16676614	.37541246	
Y01:_cons	-.00132413	-.00401958	0	.02053219	0	-.09805226	-.11151306	-.03479041	.28428383

Abbreviation: DLQI = Dermatology Life Quality Index.
Note: Diagonal elements represent coefficient variances; off-diagonal elements represent covariances. Used to derive standard errors and assess potential multicollinearity. Age = respondent's age in years. Sex and severity4 are categorical variables expanded into dummy indicators, where "1b" denotes the reference category and "2", "3", and "4" represent additional categories. *_cons* indicates the model intercept.

Interpretation of the Variance-Covariance Matrix

Variance (diagonal elements)

These values describe the uncertainty associated with each estimated coefficient.

- DLQI variance: moderate size → indicates that the DLQI effect is estimated with reasonable precision.
- Age variance: very small → the age coefficient is estimated very precisely, although its effect size was near zero.
- Sex category variance (dummy variables): one sex variable shows substantial variance → the sex effect is estimated imprecisely, likely due to limited variation between groups.
- Severity category variance (dummy variables): highest variances overall → the severity effects carry considerable uncertainty, which is common when subgroup sizes are small or imbalanced.
- Intercept variance: large variance → expected, as the intercept captures baseline variability in the model.

Covariance (off-diagonal elements)

These indicate relationships between coefficient estimates.

- Most covariances are extremely close to zero, indicating that predictors do not share strong linear relationships within the model.
- Covariance between DLQI or age and other predictors is minimal, showing no evidence of multicollinearity.
- Sex and severity dummy variables show only small covariances, suggesting limited overlap in the information they contribute.
- Severity categories correlate mildly with each other, which is expected because they stem from an ordinal classification.

Overall conclusion

- DLQI and age coefficients are estimated with the highest precision.
- Sex and severity effects show greater uncertainty, consistent with small category sizes or low variability.
- The covariance values are uniformly small, meaning the **model does not exhibit signs of multicollinearity.**
- The variance–covariance structure supports **stable estimation** and is consistent with the model's reported standard errors and inference.

Code **(key steps- whole code can be provided upon request)**

```
* 0) Keep complete cases
keep if !missing(EQ5D5L, DLQI, age, sex, severity4)

* 1) Rescale EQ-5D-5L to (0,1) if utilities include negative values
scalar eps = 1e-6
quietly summarize EQ5D5L
scalar umin = r(min)
scalar den  = (1 - umin) + 2*eps

gen double Y01 = (EQ5D5L - umin + eps) / den
replace Y01 = eps     if Y01 <= 0
replace Y01 = 1 - eps if Y01 >= 1

* 2) Fractional logit model (linear DLQI only)
fracreg logit Y01 DLQI age i.sex i.severity4, vce(robust)

* 3) Variance–covariance matrix
matrix V = e(V)
matrix list V

* 4) Back-transform predictions (optional check)
predict double YHAT01
gen double EQ5D5L_hat = YHAT01*den + (umin - eps)

* 5) Goodness-of-fit checks
summarize EQ5D5L EQ5D5L_hat
corr EQ5D5L EQ5D5L_hat
estat gof

* 6) Marginal effects
margins, dydx(DLQI age) atmeans
margins i.severity4
```
